# Spatial omics: Navigating to the golden era of cancer research

**DOI:** 10.1002/ctm2.696

**Published:** 2022-01-18

**Authors:** Yingcheng Wu, Yifei Cheng, Xiangdong Wang, Jia Fan, Qiang Gao

**Affiliations:** ^1^ Center for Tumor Diagnosis & Therapy and Department of Cancer Center Jinshan Hospital and Jinshan Branch of Zhongshan Hospital Zhongshan Hospital Fudan University Shanghai 200540 China; ^2^ Department of Liver Surgery and Transplantation and Key Laboratory of Carcinogenesis and Cancer Invasion (Ministry of Education) Liver Cancer Institute Zhongshan Hospital Fudan University Shanghai China; ^3^ Department of Pulmonary and Critical Care Medicine Zhongshan Hospital Institute for Clinical Science Shanghai Institute of Clinical Bioinformatics Shanghai Engineering Research for AI Technology for Cardiopulmonary Diseases Jinshan Hospital Centre for Tumor Diagnosis and Therapy Fudan University Shanghai Medical College Shanghai China; ^4^ Key Laboratory of Medical Epigenetics and Metabolism Institutes of Biomedical Sciences, Fudan University Shanghai China; ^5^ State Key Laboratory of Genetic Engineering Fudan University Shanghai China

**Keywords:** cancer ecology, single‐cell RNA‐seq, spatial omics, tumour microenvironment

## Abstract

The idea that tumour microenvironment (TME) is organised in a spatial manner will not surprise many cancer biologists; however, systematically capturing spatial architecture of TME is still not possible until recent decade. The past five years have witnessed a boom in the research of high‐throughput spatial techniques and algorithms to delineate TME at an unprecedented level. Here, we review the technological progress of spatial omics and how advanced computation methods boost multi‐modal spatial data analysis. Then, we discussed the potential clinical translations of spatial omics research in precision oncology, and proposed a transfer of spatial ecological principles to cancer biology in spatial data interpretation. So far, spatial omics is placing us in the golden age of spatial cancer research. Further development and application of spatial omics may lead to a comprehensive decoding of the TME ecosystem and bring the current spatiotemporal molecular medical research into an entirely new paradigm.

## Graphical abstract

1. Spatial omics is transforming our understanding of the cancer ecosystem at the systemic level.
2. The integration of spatial omics and single‐cell omics can fundamentally improve our understanding of tumourigenesis and cancer microenvironment.
3. Generating the spatial atlas of human cancers across multiple omics and timescales will potentially pioneer the revolution of spatiotemporal molecular medicine.

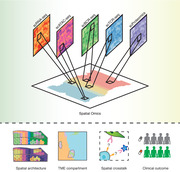

## 1 BACKGROUND

One of the central issues that hinders successful anti‐cancer treatment is the heterogeneity of tumour microenvironment (TME).^1^ Spatially, TMEs in distinct tumours are diversely organised and hierarchically structured.^2^, ^3^. Shapes of TMEs are critical for cancer cell fate determination and development, which are coordinated by precise tumour intrinsic transcriptional regulation and intercellular crosstalk.^4^, ^5^, ^6^ In response to external stimuli (e.g., chemotherapy), spatial reprogramming will be initiated including anti‐tumour immunity renaissance and stromal cell relocation.^7^ Understanding the spatial structure of TME assembly is hence essential for discovering tumourigenesis mechanisms and designing novel therapeutic strategies.

A challenge in decoding tumour spatial structure is how to capture the high‐throughput spatial profile of TME at the genome‐wide level. Solving this issue requires the ability to record the transcriptional information and spatial coordinates simultaneously. An applicable way is high‐dimensional imaging (i.e., imaging mass cytometry, IMC, and multiplex immunohistochemistry),^8^, ^9^; however, those approaches can only quantify the low‐throughput profile of TME (designed gene sets) rather than the whole transcriptome/proteome.

By contrast, the state‐of‐the‐art spatial omics has now made the whole transcriptome or even epigenome measurable.^10^, ^11^, ^12^ In light of this, we review the technological advances plus computational strategies of spatial omics and discuss how they may accelerate spatiotemporal oncological research. The clinical relevance of spatial omics will potentially extend into novel clinical‐relevant biomarker discovery, novel immunotherapy designing, and precision medicine. The ultimate spatial tumour atlas will be an essential resource uncovering the black box of cancers across space and time.

## 2 SPATIAL OMICS TECHNOLOGIES

### 2.1 Laser capture microdissection‐based approaches

**FIGURE 1:**
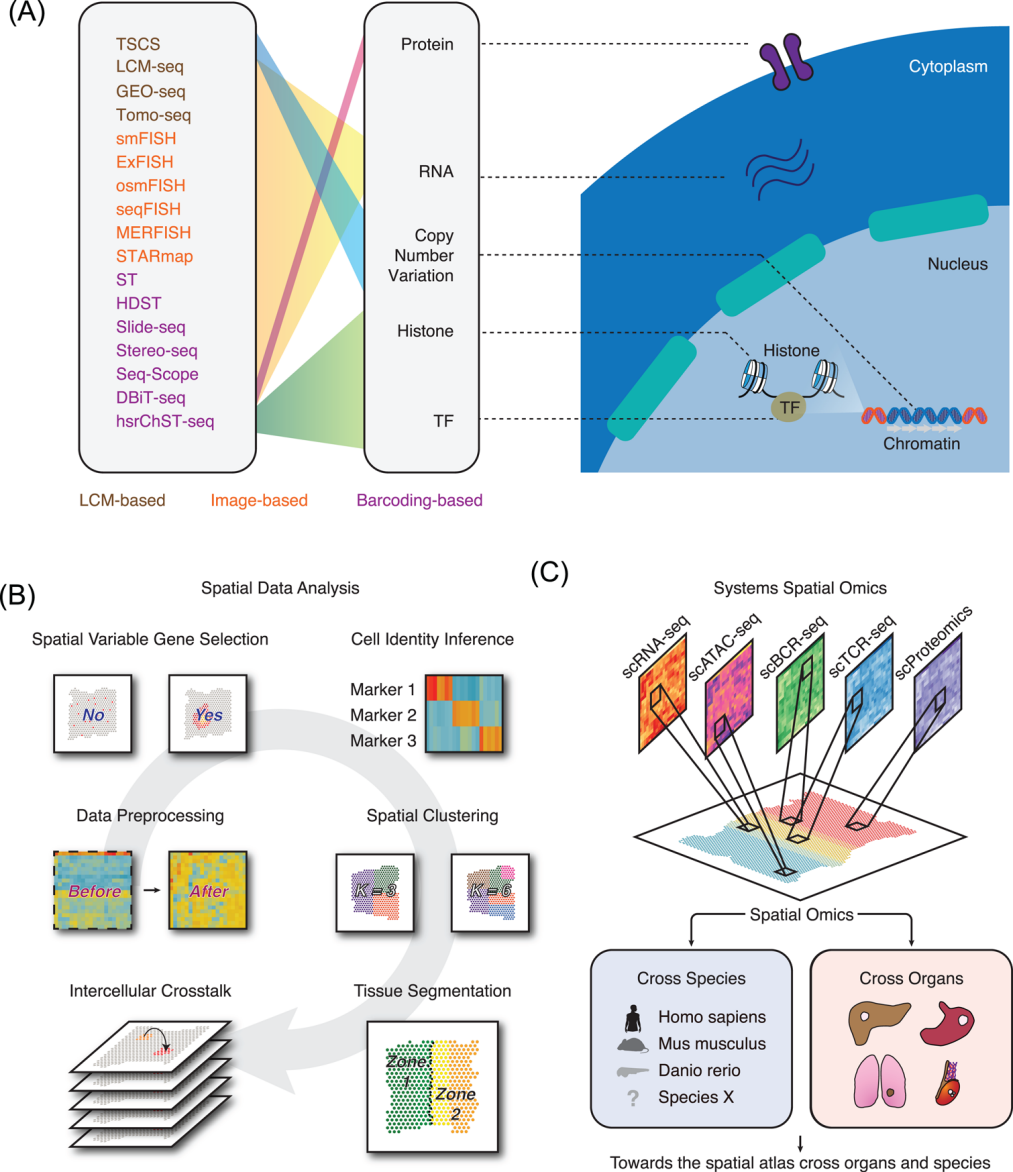
Spatial omics can decode the three‐dimensional structure of tumour microenvironment. (A) Summary of published spatial omics technologies. The brown text represents the LCM‐based technologies. The orange text represents the imaging‐based technologies. The purple text represents the barcoding‐based technologies. TF, transcription factor; LCM, laser capture microdissection. (B) The data analysis strategies which can be adopted in spatial omics data treatment. (C) Spatial omics can be utilised to study cancer samples across different species and distinct organs. Integrating of spatial omics and other omics techniques can systematically decode the structure of tumour microenvironment. scRNA‐seq, single‐cell RNA‐seq; scATAC‐seq, single cell assay for transposase‐accessible chromatin‐seq; scBCR‐seq, single cell B‐cell receptor‐seq; scTCR‐seq, single‐cell T‐cell receptor‐seq; mIHC, multiplex immunohistochemistry

The first attempt to dissert the high‐throughput spatial tissue structure can be traced to laser capture microdissection (LCM)‐based strategies^13^, ^14^ (Figure 1A). This approach utilises LCM to dissect tissues into small segments which are subsequently profiled using high‐throughput technologies such as RNA‐seq. For example, LCM‐seq^14^, ^15^, ^16^ combines single‐cell RNA‐seq (scRNA‐seq) and LCM to trace the spatial transcriptome at the single‐cell level. This technology allows the accurate quantification of compartment of tissue structures and the discovery of diversified cell subpopulation distribution within tissues. Using a similar strategy, topographic single cell sequencing (TSCS) is designed to capture the genomic copy number profile of single tumour cells spatially.^17^ By utilising TSCS in breast cancer samples, the results show a direct genomic lineage of breast cancer cell progression. Interestingly, the authors observe that most mutations and copy number aberrations evolved prior to invasion, indicating that cancer cells are well prepared before progression. This technique allows the unbiased discovery of copy number variations at the 2D level. Geographical position sequencing (GEO‐seq),^18^, ^19^ another method combining the two technologies (LCM and scRNA‐seq), can capture the spatial transcriptome based on a relatively small number of cells. However, compared with LCM‐seq, each spot of GEO‐seq captures more number of cells. Similarly, Tomo‐seq enables the cryosectioning of the region of interest and allows the RNA‐seq on individual sections.^20^, ^21^, ^22^ In the context of oncology, only a few spatial omics studies utilised LCM‐based approaches.^14^, ^23^ To summarise, LCM‐based high‐throughput technologies can quantify the transcriptome at the cellular level, however, those technologies failed to reach higher resolution and can merely trace the regional location information. Laser microdissection is also time‐consuming, posing challenges for capturing the high‐throughput profile of complex tissues without spending a lot of time.

### 2.2 Image‐based in situ transcriptomics

Another strategy to capture the spatial architecture of tissue transcripts is image‐based in situ transcriptomics technology (Figure 1A). Single‐molecule fluorescence in situ hybridization (smFISH)^24^, ^25^, ^26^, ^27^, ^28^ can detect several RNAs at the same time. On the basis of smFISH, expansion FISH (ExFISH), ouroboros single‐molecule FISH (osmFISH), and sequential FISH+ (seqFISH+) are designed to increase the number of detected genes (up to 10 000).^29^, ^30^, ^31^, ^32^, ^33^, ^34^ Multiplexed error‐robust fluorescence in situ hybridization (MERFISH), a robust single‐molecule imaging approach, can capture 100–1000 distinct RNA species in hundreds of individual cells,^35^ which evolves the gene throughput to ∼10000 in 2019.^36^ Similarly, Spatially‐resolved Transcript Amplicon Readout mapping (STARmap), integrating hydrogel‐tissue chemistry, targeted signal amplification, and in situ sequencing, is tested to capture 160–1020 genes simultaneously.^37^ Those imaged‐based technologies identified specific RNAs enriched in cellular compartments or even high‐order chromatin structure.^36^, ^38^ In a nutshell, most image‐based in situ transcriptomics technologies cannot capture the whole transcriptome profile but can offer single‐cell or even subcellular resolution within tissues, hence enabling the discovery of complex cellular states of cancer cells.^39^, ^40^

### 2.3 Spatial barcoding‐based transcriptomics

Distinct from image‐based in situ transcriptomics, spatial barcode‐based approaches allow the unbiased sequencing of RNA species at the whole transcriptome level (Figure 1A). Spatial transcriptomics (ST, also named as Visium), one of the most widely used spatial omics technologies, enables the sequencing of 6 mm × 6 mm tissues with each spot at the resolution of ∼100 μm containing 2–10 cells.^41^ One of the advantages of such a method is that it can capture thousands of genes with low‐level transcript expression and even from formalin‐fixed paraffin embedding (FFPE) tissues.^42^ The upgraded method named high‐definition spatial transcriptomics (HDST) was then developed with resolution at 2 μm.^43^ This approach opens the avenue of spatial quantification of complex tissues at the single‐cell resolution. Slide‐seq^44^ and Slide‐seqV2^45^, another high‐resolution spatial sequencing technology, can reach the ∼50% RNA capture efficiency of scRNA‐seq and successfully characterise the spatiotemporal developing trajectory of mouse neocortex. Spatio‐Temporal Enhanced REsolution Omics‐sequencing (Stereo‐seq), which combines DNA nanoball chips and in situ RNA capture, can reach the resolution ∼0.5 μm of each bin.^46^ Seq‐Scope, newly‐developed spatial transcriptome sequencing technique based on a solid‐phase amplification of randomly barcoded single‐molecule oligonucleotides, can also reach the sub‐cellular resolution (0.5–1 μm). Those methods put us in a unique position in exploring the new functions of organelles and may lead to a major advance of our understandings of spatiotemporal molecular medicine.^47^, ^48^

Rather than placing the spatial barcodes onto chips, a new class of quantifying spatial coordination of RNAs, named microfluidic deterministic barcoding, was recently developed in Rong Fan's lab. Using a unique barcode delivering method, deterministic barcoding in tissue for spatial omics sequencing (DBiT‐seq) does not require sophisticated steps of tissue lysis to release mRNAs but presents high resolution at ∼10 μm.^49^, ^50^ On the basis of this work, they developed spatial epigenomics technique for histone modifications quantification such as H3K27me3, H3K4me3 and H3K27ac.^51^ This fantastic method enables the discovery of spatially key regulatory elements controlling identity (i.e., spatial enhancer prediction) and brings the epigenetics research into the spatial era. This microfluidic deterministic barcoding based approach promises to extend single omics to spatial multi‐omics sequencing and will open exciting opportunities in quick and straightforward profiling of complex cells and tissues. The revolution of the above spatial omics will pave the way for spatiotemporal molecular medical research^47^, ^48^ and refresh our understanding of the single‐cell heterogeneity and spatial diversity in TME (Figure 1A).

### 2.4 Spatial proteomics

The surging of spatial proteomics allows the detection of dozens of proteins without losing the spatial location. Mass spectrometry‐based method is one of the highly multiplex techniques to capture the protein spatial intensity. Multiplexed ion beam imaging (MIBI), using secondary ion mass spectrometry to image labelled antibodies, is able to analyse one hundred markers of the same tissue.^52^ This technology yields precise quantification of immune cell subpopulation^53^ and their spatial patterns^54^ inside the tumour. IMC is another method dependent on metal‐tagged antibodies and enables the imaging of over 100 antibodies.^55^ Such method offers unprecedented opportunities to explore regional immunity composition and topological function units of TME.^56^ Another technology named CO detection by indexing (CODEX) is capable of profiling up to 50 proteins of single slide based on imaging antibodies conjugated to barcodes.^57^ However, all those methods are dependent on the performance of antibodies and are relatively costly. It is still challenging to increase the current throughput to proteome‐wide. Bias may also exist when designing the panel of markers rather than discovering functional proteins from the proteomics data.

### 2.5 Spatial metabolomics

Identifying the difference of metabolites and the spatial organization of tissues is essential to decipher intra tumour heterogeneity and understand the cancer systems profoundly, however, the spatial metabolic features of tumour s are largely unclear. Matrix‐assisted laser desorption ionization imaging mass spectrometry (MALDI‐IMS) allows the detection of metabolites without losing the spatial information.^58^, ^59^ Desorption electrospray ionization (DESI)‐IMS is another method to detect the spatial dynamics metabolites without destroying the tissues.^60^, ^61^, ^62^, ^63^ Airflow‐assisted desorption electrospray ionization (AFADESI)‐IMS^64^ further advances the multiplex capacity and can cover 1,500 metabolites. Applying such method in profiling esophageal squamous cell carcinoma showed the spatial tiny structure of metabolites including pyrroline‐5‐carboxylate reductase 2 (PYCR2) and uridine phosphorylase 1 (UPase1).^65^ With the coming of the spatial omics era, spatial metabolomics will become a useful toolkit for identifying novel disease signatures.

### 2.6 Spatial multi‐omics technologies

Cancer is a multifactorial disorder associated with multiple genetic and environmental factors; hence jointly dissecting spatial multi‐omics profile may enable us to reconstruct the key processes of tumourigenesis. DBiT‐seq supports recording the spatially barcoded mRNA and profiling proteins of interest (a panel of 22 proteins) at the same time.^49^ Deterministic barcoding, the underlying key mechanism of this technology, allows the accurate delivering of barcodes containing spatial coordinates without tissue dissociation. The development of spatial multiomics (SM‐Omics) also offers integrated and ST and antibody‐based proteomics profiling without the need for sophisticated infrastructure.^66^ This platform enables the automatic profiling of 96 samples and efficiently generates the combined RNA and protein profile in ∼2 days. By testing this platform in mouse brain cortex samples, significant correlation between specific mRNA and protein expression is observed. The authors further develop a computational pipeline and claim its easy deployment to the wide scientific community.

Another strategy to simultaneously decode the spatial and cellular dynamics is to *in silico* integrating spatial‐omics and single‐cell omics data. Spatially‐resolved Transcriptomics via Epitope Anchoring (STvEA) enables the enrichment of multiplex immunohistochemistry data with scRNA‐seq^67^ and is also designed to map CODEX imaging data to scRNA‐seq. As for the epigenomic data, another group imputed single cell multiome (chromatin accessibility and transcriptome multi‐omics) profile from the Visium ST data.^68^ Such analysis predicted the spatial epigenetics activity of key genes and linked the opening motif dynamics with phonotypes. These strategies can be potentially adopt to other spatial omics technologies (i.e., spatial metabolomics^65^). In general, capturing/inferring the spatial multi‐omics profile simultaneously is still in its infancy. The next step is to extend existed single‐omics to multi‐omics and enable the jointly profiling of the complex system of TME.

## 3 COMPUTATIONAL METHODS FOR SPATIAL OMICS

While the complexity of spatial omics data is what makes it powerful, it also makes them hard to interpret. Usually, these data are collected in batches and generated in large quantities. The large amount of data pose great challenges for computational biologists to digest the big data and construct computational pipelines. Here, we review recent computational methods spanning from data preprocessing, spatial variable gene selection, spatial clustering and tissue segmentation, to spatial inter‐cellular crosstalk (Figure 1B and Table 1).

**TABLE 1:** Summary for algorithms designed for spatial omics analysis

**Name**	**Usage**	**Environment**	**URL**
SCTransform	Data preprocessing	R	https://github.com/ChristophH/sctransform
Giotto	Data preprocessing, spatial variable gene identification, cell identity inference, cell–cell crosstalk modelling, clustering analysis	R	http://spatialgiotto.rc.fas.harvard.edu/giotto.html
Seurat	Data preprocessing, spatial variable gene identification, cell identity inference, clustering analysis	R	https://satijalab.org/seurat/vignettes.html
SpatialDE	Spatial variable gene identification	Python	https://github.com/Teichlab/SpatialDE
trendsceek	Spatial variable gene identification	R	https://github.com/edsgard/trendsceek
scGCO	Spatial variable gene identification	Python	https://github.com/WangPeng‐Lab/scGCO
SPARK	Spatial variable gene identification	R	https://github.com/xzhoulab/SPARK
SOMDE	Spatial variable gene identification	Python	https://pypi.org/project/somde/
BayesSpace	Clustering analysis	R	http://www.bioconductor.org/packages/release/bioc/html/BayesSpace.html
SpatialCPie	Clustering analysis	R	https://www.bioconductor.org/packages/release/bioc/html/SpatialCPie.html
SPOTlight	Cell identity inference/deconvolution	R	https://github.com/MarcElosua/SPOTlight
RCTD	Cell identity inference/deconvolution	R	https://github.com/dmcable/RCTD
stereoscope	Cell identity inference/deconvolution	Python	https://github.com/almaan/stereoscope
DSTG	Cell identity inference/deconvolution	Python	https://github.com/Su‐informatics‐lab/DSTG
STUtility	Data preprocessing, spatial variable gene identification, clustering analysis, tissue segmentation, image processing	R	https://ludvigla.github.io/STUtility_web_site/index.html
Squidpy	Data preprocessing, spatial variable gene identification, cell identity inference, cell–cell crosstalk modelling, clustering analysis, tissue segmentation	Python	https://github.com/theislab/squidpy
Baysor	Tissue segmentation	Linux	https://github.com/kharchenkolab/Baysor
SPATA	Tissue segmentation, trajectory modelling	R	https://themilolab.github.io/SPATA/index.html
stLearn	Trajectory modelling; cell–cell crosstalk modelling	Python	https://stlearn.readthedocs.io/en/latest/
GCNG	Cell–cell crosstalk modelling	Python	https://github.com/xiaoyeye/GCNG
SpaOTsc	Cell–cell crosstalk modelling	Python	https://github.com/zcang/SpaOTsc
MISTy	Cell–cell crosstalk modelling	R	https://github.com/saezlab/mistyR

### 3.1 Data preprocessing

The first crucial step for spatial data downstream analysis is normalization against the sequencing depth. The variance of RNA read counts of each spot can be diverse partly due to heterogeneous cell type composition. To resolve this question, several algorithms have been designed for scRNA‐seq, and some of them are still suitable for ST data analysis such as SCTransform.^69^ This algorithm integrates regularised negative binomial regression and Pearson residuals which proved to be fit for ST data.^54^ SCTransform was embedded in Seurat toolkit and is very easy‐to‐use.^70^ Another package supporting spatial RNA read normalization is Giotto.^71^ This toolkit supports the all‐in‐one data preprocessing functions including library size adjustment, log transformation and data scaling. Those methods are broadly applicable for spatial barcoding‐based transcriptomics such as ST or Slide‐seq. However, the majority of those computational algorithms do not consider the spatial coordinates and therefore do not measure spatial variability. Spatial normalization and scaling was critical for improving the performance of spatial omics (e.g., normalised expression is more linked with specific morphologies and capture cell types’ relative proportions^72^). Applying the noise reduction algorithm in image processing such as smoothing or wavelet transform may also enhance the performance of spatial omics.

### 3.2 Spatial variable gene identification

When data normalization is completed, a question naturally arises: are there spatially variable genes linked with the well‐organised tissue structures? For example, some genes show extremely variable spatial expression patterns (i.e., tumour and normal edge specific genes), while some genes are ubiquitously expressed (i.e., cancer housekeeping genes). Computationally, quantifying the spatial variable genes is hence expected to be fundamental to discovering the molecular basis of TME architecture.^73^ The direct way is to compute the differentially expressed genes according to clusters/anatomical structures^70^ or select genes with high variance.^71^ Those methods partly depend on prior knowledge (e.g., Pathologist's annotation) and supervised clustering results. On the contrary, spatial‐patterned‐based methods do not rely on supervised annotation and is broadly applicable for spatial data. Seurat^70^ utilised variogram models and measures the distance between two spots. SpatialDE builds on Gaussian process regression and decomposes gene expression into spatial and non‐spatial elements.^73^ Another group reported the marked point process‐based statistical framework which proved to be robust for simulated and real data^74^ This algorithm is non‐parametric and does not depend on any prior knowledge. scGCO, a method based on Markov Random Fields with graph cuts, is able to process millions of cells in hours but does not require consuming large memory. Interestingly, scGCO is widely applicable to a full range of spatial data even image‐based in situ transcriptomics (e.g., seqFISH and MERFISH).^75^ SPARK is also a time‐saving algorithm^76^. The authors use the generalised linear spatial models and provide efficient control of type 1 errors. Recently, Hao et al. proposed an artificial neural network (ANN) based clustering method named SOMDE and utilised Gaussian process to conduct feature selection.^77^ This method is also featured with super‐fast speed (∼5 min for 20 K spots) and low memory consumption. In summary, a wide spectrum of algorithms are focused on spatial variable gene identification, partly due to its importance for selecting features for downstream spot clustering and dimensional reduction analysis. Most of them are designed for Visium. Hence, their performance on other techniques (e.g., DBiT‐seq and MERFISH) still remain unknown due to the completely different read count distributions and spot resolutions.

### 3.3 Clustering the spots

Defining the clusters in a given tissue is fundamental for cell‐type identification and downstream functional annotation. In the context of scRNA‐seq, filtering the pure clusters, the population exhibiting identical functions, requires manual gene signature checking and tricky parameter adjustment. A robust method is to compute the entropy metric (ROGUE algorithm^78^) or Gini index (GiniClust algorithm^79^, ^80^) of given clusters. As for spatial data treatment, simultaneously considering the locational information and mixed cell types pose challenges for accurate spot clustering. The direct way is to provide a user‐friendly package for users to manually select the parameters such as resolution.^81^ More complicatedly, Zhao et al. utilised the Bayesian model with a Markov random field and successfully enhanced the clustering efficacy.^82^ This algorithm has been demonstrated to perform well on multiple datasets (i.e., squamous cell carcinoma and prefrontal cortex) generated by distinct spatial omics technologies. Other computational methods utilising graph convolutional network or deep learning also appeared to perform well for ST data.^83^, ^84^. To summarise, the existing clustering methods are mostly designed for scRNA‐seq and do not consider the neighbourhood spot information or spatially patterned structure, pressing the need for developing more algorithms. Robust spatial clustering algorithms can generate the precise subpopulation maps of cancer cells and correlate the cellular states with their spatial distributions.

### 3.4 Cell identity inference

A lot of strategies have emerged to link gene expression with cell identity, although most of them are designed for single cell transcriptomics.^85^ Some of them are based on the correlation between test data and reference data, while others require supervised classification and classifier training.^85^ Given the spatial omics data usually contain a mixture of cells such as ST, new algorithms are needed for optimising the automatic analysis pipeline. One of the most used approaches is deconvolution, such as SPOTlight (using non‐negative matrix factorization regression),^86^ RCTD (using non‐negative least‐squares regression),^87^ DSTG (using graph‐based convolutional networks)^88^ and stereoscope (a probabilistic model based on the negative binomial distribution).^89^ Another practical way is to modify the scRNA‐seq integration algorithm. For example, Seurat^70^ enabled the ‘anchor’‐based workflow and transferred labels from scRNA‐seq data to ST data. This algorithm was previously demonstrated effective for cross‐species integration and has now shown robustness in ST. The above methods are mostly designed for spatial omics of near‐single‐cell resolution such as Visium and can effectively infer the cellular composition of a spot mixture. Also, those algorithms largely rely on matched scRNA‐seq data to compute the cell‐type probability. As a result, such analysis is largely dependent on single‐cell clustering analysis and the uncertain level of resolution parameter. Annotating higher resolution data (e.g., Stereo‐seq) without prior knowledge is still challenging.

### 3.5 Single‐cell segmentation

Accurately defining the tissue borders is critical for understanding how cells locally interact with others (i.e., cancer cells interact with immune cells). For example, robust segmentation is able to increase the number of detected cells and allow the inference of the real cell states.^90^ To resolve this question, Baysor enables cellular segmentation based on the likelihood of transcriptional composition, size and shape of the cell.^90^ A pipeline named Squidpy allows the extraction of image features and nuclei segmentation.^91^ This pipeline is largely based on image processing and provides options of different deep learning algorithms such as Stardist^92^ and Cellpose.^93^ However, a lot of questions regarding tissue segmentation still remain unexplored. How to computationally model the cellular spatial patterns such as immune cells (sparse) and endothelial cells (linear)? How to segment the organelle structure (e.g., nuclei and cytoplasm) and trace the RNA origin in subcellular spatial omics data? A possible way is to train a machine learning model by using the spatial coordinates of existed multiplexed immunofluorescence imaging, given that spatial distribution patterns of spatial omics is similar to immunofluorescence. New algorithms supporting high spatial resolution segmentation need to be developed and will allow its broad application in the spatial omics community.

### 3.6 Cell–cell communication analysis

In the context of scRNA‐seq data analysis, decoding cell–cell communication is crucial for understanding how cells interact with each other and how such crosstalk networks are changed under specific disease conditions such as cancer.^94^ Decoding the global cell–cell communication network will help design specific targeting strategies.^6^, ^95^ Existed algorithms^96^, ^97^, ^98^, ^99^ are mostly designed for scRNA‐seq and do not consider spatial information. To trace the spatial cellular communication dynamics, several algorithms have been proposed. For example, SpaOTsc can infer intercellular gene–gene regulatory information flows between genes by using a machine learning model.^100^ Another intercellular communication quantification package named stLearn can compute the morphological similarity between spots and then perform the cell–cell communication analysis.^101^ GCNG encodes the spatial location as a graph, integrates it with an expression profile using supervised training, and infers the cell–cell interactive scores.^102^ MISTyis a flexible and scalable machine learning framework for capturing the cell–cell communication score and performs well on multiple datasets.^103^ Well‐established ST pipelines including Giotto^71^ and Squidpy^91^ also support the spatial intercellular crosstalk analysis. By utilising those methods,^71^, ^100^ spatial interactive cells such as interacting astrocytes and inhibitory neurons were observed. Taking the advantage of high‐resolution spatial technologies such as Slide‐seq or Stereo‐seq, it may be possible to computationally model the dynamics of intercellular gene regulation and fundamental biological processes (i.e., phase separation).

## 4 SPATIAL OMICS IN CANCER RESEARCH: FROM BENCH TO BEDSIDE

Cancer can be viewed as a complex system.^104^ The evolution of tumour cells is like a dynamic interaction between microenvironmental/therapeutic selective forces and their intrinsic adaptive strategies to survival.^105^ With the help of the state‐of‐the‐art spatial omics, it is now possible to tackle a range of basic and fundamental questions of oncology at the systemic level. Current spatial research is spanning from multi‐omics (e.g., transcriptomics,^106^ epigenomics^50^), and multiple organs (e.g., breast cancer,^43^ melanoma^107^), to multiple species (e.g., Homo sapiens,^54^ Danio rerio^108^) (Figure 1C).

### 4.1 Spatially decoding the heterogeneity of TME

The core problem hindering patients’ long‐term survival is cancer heterogeneity.^1^ This heterogeneity was featured as mixed cell types with spatial differences in gene expression.^4^, ^109^ As a consequence, diverse cellular populations exist within the TME, making any gene‐ or pathway‐specific therapy less effective.^1^ Existed single‐cell omics techniques have decoded the intra tumour heterogeneity at the systems level,^110^, ^111^, ^112^, ^113^ however, such a method does not retain the spatial coordinates of each cell. In 2018, Lundeberg's lab first utilised ST to explore the prostate TME diversity in the cancer research.^106^ They precisely computed the dynamic gene expression changes during cancer progression and demonstrated the cancer cell state difference between the TME periphery and centre. In stage III metastatic melanoma samples, they found that immune‐related genes such as HLA genes and CD74 are highly active in cancer regions.^107^ Surprisingly, a recent study indicates that cancer hallmark pathways are specifically activated even in specific regions of tumours.^114^

Another grand challenge in tumour ecology research is the spatial quantification of immune cells. Andersson et al. used the deconvolution algorithm^89^ and inferred the region‐specific enrichment/depletion of B cells. They further developed a gene signature of tertiary lymphoid structure (TLS) and observed the activation of cell activation/differentiation pathway in TLS^115^. By using the SPOTlight deconvolution algorithm,^86^ the authors spatially trace the T cell subsets in glioblastoma patient samples and infer the trajectory of those T cells.^116^ We recently reported the spatiotemporal immune profile of colon cancer liver metastasis and observed the increased infiltration of MRC1^+^ CCL18^+^ macrophages in the metastatic sites.^95^ All these studies are partly based on ST which cannot directly profile the transcriptome of single immune cell but the combined profiles of cancer/immune cell mixture. In the future, more data generated by high‐resolution spatial omics (e.g., DBiT‐seq) will identify the mechanisms that are shared among varied cell‐type compositions and will help develop new therapeutic approaches targeting the spatial TME organization.

### 4.2 Tumour invasive margin: the main battlefield in the fight against cancer?

**FIGURE 2:**
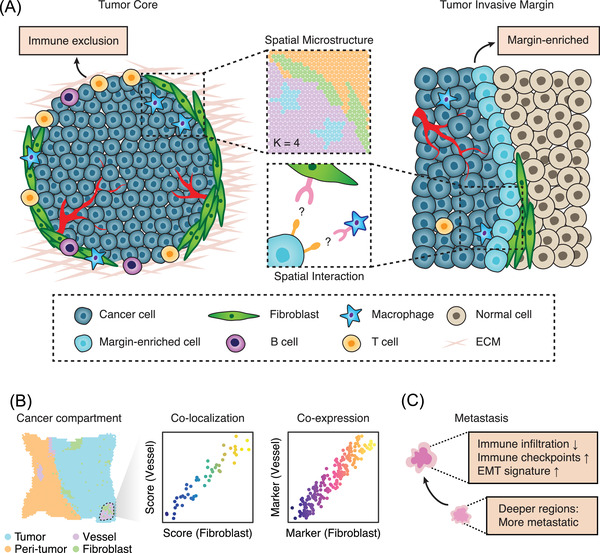
The cancer microenvironment spatial structure and compartment revealed by spatial omics. (A) The distinct microenvironment structure of tumour core and tumour margins. ECM, extracellular matrix. (B) Spatial omics can precisely capture the TME compartment such as CAFs and vessel. CAFs, cancer associated fibroblasts. (C) The microenvironment pf primary and metastatic tumour s are largely different revealed by spatial studies

Not all cancer cells are created equal. As for cancer cells located in the boundary or core regions, they exhibit different phenotype states^117^ and distinct microenvironmental features.^118^ Previous immunohistochemistry‐based low‐throughput studies^119^ largely rely on the selection of region of interest and the design of protein panels. By contrast, spatial omics allows the unbiased discovery of key cell types and genes controlling the fate of tumourigenesis. For example, by integrating ST and multiplexed ion beam imaging, Ji et al. identified tumour‐specific keratinocytes (TSKs) which are specifically located in the fibrovascular niche at leading edges.^54^ Such TSKs are associated with a high density of intercellular crosstalk with immune cells. Interestingly, this cellular population is featured with an oncogenic transcriptional program sharing the activation of HDAC1 and ETS1, indicating the potential epigenetics reprogramming resides in the leading edge zones. A zebrafish‐based study also reveals that unique edge cell states at the melanoma boundary are associated with upregulated ETS‐family transcription factor activity.^108^ These transcriptional states are validated in 10 patient samples, implicating the potential conservation across species. All those pieces of evidence place the tumour leading edges in the limelight and naturally raise the hypothesis TME conditions (i.e., tumour boundary barrier vs. tumour core) may be spatially different (Figure 2A).

### 4.3 The compartment of TME for supporting tumour growth

Significant progress has been made in the field of single‐cell omics and we now know that TME is well‐structured with immune cells, cancer‐associated fibroblasts, and the extracellular matrix,^6^, ^120^ but the challenges still remain. Importantly, it is difficult to trace back the spatial cellular states and developmental states. The evolution of spatial omics technologies helps to open the black box of TME. For example, the prostate cancer tissues can be divided into compartments with distinct gene expression profiles and pathway activities.^106^ A subset of stroma cells are specifically reactive to nearby cancer cells, while the others show inflammatory features. Inside the TME of pancreatic cancer samples, the structure of the stroma is also highly ordered. In particular, the inflammatory fibroblasts and endothelial cells are co‐localised in this region (Figure 2B). As for immune cells, their distribution is closely linked with the compartment zones.^23^, ^121^ Combined scRNA‐seq and ST revealed the pancreatic cancer subtype‐specific compartment reprogramming in response to neoadjuvant chemotherapy.^7^ Collectively, those data explain how the anatomical molecular profile of the TME compartment determines the tumourigenesis fate and how cancer cells spatially respond to therapy.

### 4.4 Tracing the spatiotemporal evolution of cancer cells

**FIGURE 3:**
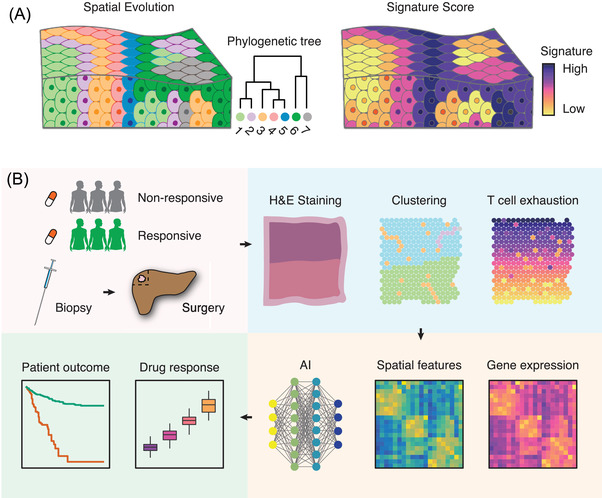
The spatial evolution of cancer cells and the potential clinical application of spatial omics. (A) Spatial genomics and transcriptomics allows the discovery pf evolution and their specific signature of cancer cells. (B) Spatial omics can be potentially used to predict the drug response and the clinical outcomes in the clinical setting. EMT, epithelial‐to‐mesenchymal transition; CAF, cancer‐associated fibroblasts

Evolution is the driving force behind cancer cell resistance or metastasis.^122^, ^123^ Previous research largely relied on multi‐region sample collection,^124^ where directly modelling the spatial evolutionary routes cell‐by‐cell was far from applicable. Now, with the help of spatial omics, tracing cancer evolution at different space coordinates and cellular units is now possible. Sundar et al. utilised the NanoString transcriptomics profiling (composed of 770 genes) and found that nearby lymph node metastases may originate from deeper subregions of the primary cancer cells.^125^ Another group reported that metastatic tumour s were depleted with immune cell infiltrates but harboured high expression of the immune checkpoints (B7‐H3, TIM‐3, etc.).^126^ Interestingly, the epithelial–mesenchymal‐transition (EMT) gradient is specifically enriched in the metastatic tumour but not the primary tumour^127^ (Figure 2C). At the genome level, recent data indicated that co‐existed clones surprisingly have different transcriptional and immunological features. Such spatial clonal diversity is deeply impacted by resident tissue structures. TSCS profiling allows the genomic lineage tracing between distinct tumour subpopulations^17^ (Figure 3A). These results highlight the spatial rearrangement during cancer evolution, and further multidimensional spatial analysis will allow a broad range of spatiotemporal molecular medicine^47^, ^48^ problems to be solved.

### 4.5 Is spatial omics still far from the clinical application?

Initial data showed that spatial arrangements of TME quantified by mIHC perform better in predicting immunotherapy response comparable to existed methods (i.e., PD‐L1 expression, immunohistochemistry, tumour mutational burden and bulk gene expression profiling).^128^ These observations indicate that spatial omics can potentially provide innovative solutions for designing precision medicine strategies. Another spatial analysis of the melanoma clinical cohort reveals the spatial interaction between PD‐1/PD‐L1 and IDO‐1/HLA‐DR was tightly linked with anti‐PD‐1 clinical response.^129^ All those data revealed that spatially quantifying the TME structure might have better prognostic power than existed biomarkers. This technique may be perfectly suited to discover how TME evolves during therapy (Figure 3B) and has the potential to reveal the targetable structures (e.g., reprogramming the tumour borderlines to engineer the cold TME). However, data from retrospective/prospective clinical cohorts are still lacking.

In the clinical practice, spatial omics technology may pave the way for precision pathology. It is now possible to link the pathological images with spatial gene expression profile by using machine learning or deep learning algorithms.^130^, ^131^ These studies may enable the prediction of the transcriptomics profile based on existed H&E staining slides which may perform better than existed biomarkers. Generating the spatial atlas of a large sample of tumours will hence not only reveal new ways to improve patients’ outcomes, but also pave the way for spatiotemporal molecular medical research.^47^, ^48^

## 5 SPATIAL TUMOUR ECOLOGY: TOWARD A NEW RESEARCH PARADIGM

### 5.1 Microenvironment is an ecosystem

**FIGURE 4:**
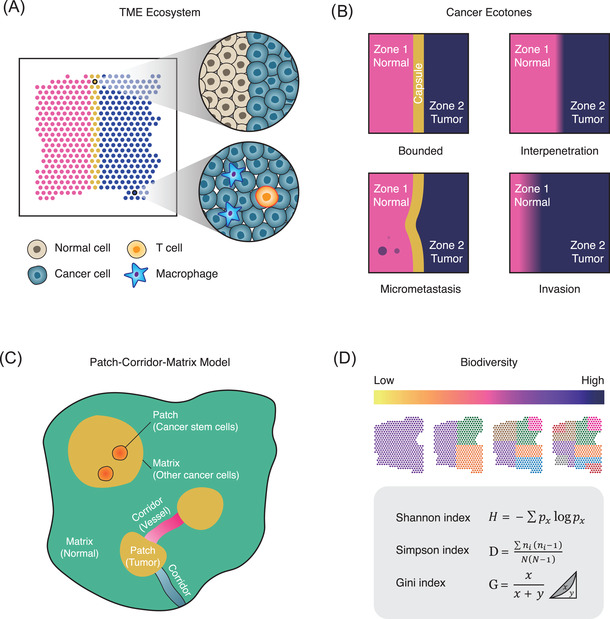
Building up the interdisciplinary link between ecology and oncology. (A) The TME is an ecosystem composed of diversified species such as immune cells and cancer cells. (B) The proposed models for describing different cancer ecotone patterns. The bounded pattern refers to equal and homogeneous interface (capsule) between tumour and adjacent normal tissues. The interpenetration pattern refers to the cancer cell infiltration into the adjacent normal tissues. The micrometastasis pattern refers to the mini cancer cell invasion into the adjacent normal tissues. The invasion pattern refers to the invasive cancer cell infiltration into the adjacent normal tissues. (C) The patch–corridor–matrix model for modelling the spatial tumour heterogeneity. (D) Methods for quantifying the spatial biodiversity inside the tumour microenvironment

The cancer microenvironment is similar to an ecological system, a mixture with distinct cellular populations and species (Figure 4A). The establishment and growth of tumours are strikingly similar to an adaptive and evolving ecosystem.^132^ For example, the species richness, which can also be referred to intra tumour heterogeneity, is potentially linked with immunotherapy robustness and patients’ long‐term outcome.^133^, ^134^ The metabolic competition between immune cells and cancer cells, which can be termed as interspecific competition, is also a key determinant of cancer progression.^135^. The behaviour of microenvironmental populations and the structure of TME can be explained by ecological theories, but more high‐dimensional data are needed.

### 5.2 Ecotones of cancer: the transitional zones of cancer‐normal communities

Edge effect refers to a greater diversity of the community at the boundary of habitats.^136^ Particularly in TME, the edge effect occurs in tumour ‐normal borders, where two distinct systems meet and mingle. Interestingly, TSKs are specifically located in the edges of skin cancer and those cells harbour frequent intercellular communications with nearby immune cells.^54^ A possible explanation is that intratumour environmental conditions drive those species to colonise habitable borders. This observation leads us to hypothesise that the edge effect in the cancer ecosystem widely exists and may be therapeutically targeted to destroy the cancer cell habitats. Those transitional zones are also termed ecotones, referring to the transition area between two biological communities (i.e., interface between forest and grass).^137^ At the boundary of TME, how the sharp border is formatted still remains unclear (Figure 4B). Does the formation of cancer ecotone help or prevent the cancer invasion? How does the transitional zone interact with immune system and forms the cold or hot tumour? The study of cancer ecotones by spatial sequencing technologies is still in its infancy. A multimodal and systems‐level spatial atlas of cancer ecotone may fundamentally improve our knowledge of cancer ecology and facilitate the designing of novel anti‐cancer strategies.

### 5.3 The patch–corridor–matrix landscape model: defining the spatial distribution patterns

The patch–corridor–matrix is an important theory for describing the spatial heterogeneity of ecological landscapes.^138^ In general, a patch refers to a spatial unit that harbours distinct features with the surrounding environment and has a certain internal homogeneity. A corridor refers to a linear structure connecting different spatial units; while a matrix refers to a continuous and widely distributed space in space. Recently published ST data reveal that the structure of tissues is in potential accordance with this model. For example, the structure of elongating/elongated spermatids is in consistency with the definition of the patch, while their progenitors such as spermatocytes and spermatogonium are located surrounding elongated spermatids which can be described as the matrix.^139^ In the context of oncology, the structure of TME and their adjacent tissues is in line with patch–matrix patterns. We hypothesise that the vascular structure conforms to corridor, which connects the patch (TME) and matrix (adjacent normal tissues). Inside the tumour, the formation of cancer stem cell niche may reside in the TME^140^, ^141^, which resembles the patch–matrix model (Figure 4C). Systematically, modelling the TME on the basis of the patch–corridor–matrix may hence provide scientific basis for understanding the size, shape, content and structure of tumour s at microscale (Figure 4C).

### 5.4 Computationally modelling the TME spatial biodiversity

Biodiversity originally refers to the biological variety of life on the Earth.^142^ Similarly, the biodiversity of cancer microenvironment is also a key factor for drug responses and patient outcomes.^134^ To accurately trace the TME diversity, statistical models are required to quantify the biodiversity of a given spatial omics data. A widely used equation is the alpha, beta and gamma diversity.^143^ Alpha diversity refers to the diversity in a particular ecosystem. This concept is usually used to measure the number of species in a given ecosystem (e.g., the species richness in TME).^143^ Beta diversity allows us to compare the biodiversity changes between ecosystems (e.g., metastatic TME vs. primary TME).^143^ Gamma diversity refers to the measure of the overall diversity in a large region.^143^ Another index assessing biodiversity is Shannon entropy index.^144^ This index represents the uncertainty that we can predict which species the individuals randomly selected in the community belong to. If the TME consists of only a single species (e.g., cancer cells), then the randomly selected individual must be that unique species. At this time, the Shannon entropy index is zero. As the number of species in the community increases, the Shannon entropy index will increase. Similarly, the Simpson index^145^ and Gini coefficient^146^ also represent species richness and evenness. The classic Simpson index represents the probability that two randomly selected individuals in the community belong to the same species. When the species richness of the community increases, this probability decreases, that is, the Simpson index decreases as the species richness increases.^145^ With the help of those biodiversity quantification indexes (Figure 4D), it is now possible to link the biodiversity with spatial cancer phenotypes such as immune exclusions.

## 6 DISCUSSION AND FUTURE PERSPECTIVES

To decode the tumour ecosystem, we need to model how individual cells work and how they interact with each other. Although high‐throughput spatial sequencing technologies coupled with the state‐of‐the‐art computational algorithms have greatly improved our understanding of tumour architecture, many pressing questions still remain to be answered, especially the profiling of the intact tissue structure. Existed experimental protocols merely enable the sequencing of tiny slide(s) of tissues. In fact, such profiling may represent the partial tissue expression profile and cannot fully capture the 3D architecture. An interesting example is the 3D transcriptomics reconstruction of the developing heart,^147^ which raises the possibility of generating the 3D model of other tissues such as tumours. Computational strategies originally designed for radiomics, such as 3D Slicer^148^, are expected to reconstruct the spatial molecular organization. We hypothesise that, with the development of advanced spatial omics technologies, a 3D bird's‐eye view of TME that encompasses transcriptomics, proteomics and metabolomics may pave the way towards the comprehensive decoding of the TME ecosystem.

We are now in the golden age for spatiotemporal molecular medicine^47^, ^48^ research. Spatial omics is transforming our understanding of cancer milieu by offering the precise spatial coordinates of cellular and molecular profiles at the systemic level^149^, ^150^, ^151^. At the same time, existed methods pose experimental and computational challenges for optimising current protocols and expanding the scope of these models. How to integrate biology data and mathematics model into the same framework, establish the interdisciplinary link between ecology and oncology, digest the booming spatial omics data and develop sophisticated analytical algorithms are still challenging. Achieving those goals are of exceptionally significance for unravelling the biology of TME as well as establishing a framework to explain the aggressive characteristics of malignant cells. Generating the spatial atlas of human cancers across multiple omics and timescales can fundamentally improve our understanding of tumourigenesis, pioneer the revolution of medical research paradigm, and ultimately facilitate the designing of advanced therapeutic strategies in the near future.

## CONFLICT OF INTEREST

The author declares that there is no conflict of interest that could be perceived as prejudicing the impartiality of the research reported.
